# Pneumonitis During Neoadjuvant Chemoimmunotherapy for Triple-Negative Breast Cancer: A Case Report

**DOI:** 10.7759/cureus.109003

**Published:** 2026-05-17

**Authors:** Athanasios Bakalis, Konstantinos Bouliaris, Fotini Psoma, Ioanna K Sgantzou, Georgia Lambrodimou

**Affiliations:** 1 Surgery, General Hospital of Larissa, Larissa, GRC; 2 Radiology, General Hospital of Larissa, Larissa, GRC; 3 Oncology, General Hospital of Larissa, Larissa, GRC

**Keywords:** immune checkpoint inhibitors, neoadjuvant therapy, paclitaxel, pembrolizumab, pneumonitis, triple-negative breast cancer

## Abstract

Triple-negative breast cancer (TNBC) is commonly treated with standard chemotherapy regimens, with immune checkpoint inhibitors (ICIs) recently incorporated into neoadjuvant therapy, resulting in improved pathological complete response (pCR) rates and survival outcomes. However, the use of ICI therapies has also raised concerns regarding potential adverse events, including pneumonitis.

In this report, we present a case of hyperacute treatment-related pneumonitis occurring shortly after pembrolizumab administration, followed by two recurrent episodes after paclitaxel re-exposure. Early discontinuation of chemoimmunotherapy followed by corticosteroid treatment led to rapid clinical improvement, followed by surgical intervention. This case highlights the diagnostic and management challenges associated with treatment-related pneumonitis in patients receiving combination chemoimmunotherapy, emphasizing the importance of early recognition and prompt intervention.

## Introduction

Triple-negative breast cancer (TNBC) is an aggressive subtype of breast cancer characterized by a lack of expression of the estrogen receptor (ER), progesterone receptor (PR), and human epidermal growth factor receptor 2 (HER2). It represents approximately 15% of invasive breast cancers and is associated with poorer clinical outcomes and prognosis compared with other subtypes [1].

Neoadjuvant chemotherapy (NACT) represents the standard of care in early-stage (T1c/N0 or greater) TNBC, aiming to reduce tumor burden and increase rates of pathological complete response (pCR), which correlates with improved survival outcomes. Taxane-based regimens, particularly paclitaxel, remain a cornerstone of treatment in this context [2].

The introduction of immune checkpoint inhibitors (ICIs) has further improved outcomes in TNBC. Pembrolizumab, an anti-programmed death 1 (PD-1) monoclonal antibody, enhances antitumor immune responses by releasing inhibitory signaling and restoring cytotoxic T-cell activity [3]. The KEYNOTE-522 trial demonstrated that the addition of pembrolizumab to NACT significantly improves pCR, overall survival, and event-free survival in patients with early-stage TNBC [4].

Despite its efficacy, pembrolizumab is associated with immune-related adverse events, including pneumonitis, a rare but potentially serious complication [5]. Paclitaxel has also been rarely associated with pulmonary toxicity, complicating attribution in patients receiving combination therapy [6].

In this report, we present a case of TNBC complicated by hyperacute and recurrent pneumonitis during neoadjuvant chemoimmunotherapy with paclitaxel, carboplatin, and pembrolizumab, highlighting the diagnostic complexity and therapeutic challenges associated with treatment-related pneumonitis in this setting.

## Case presentation

**Figure 1:**
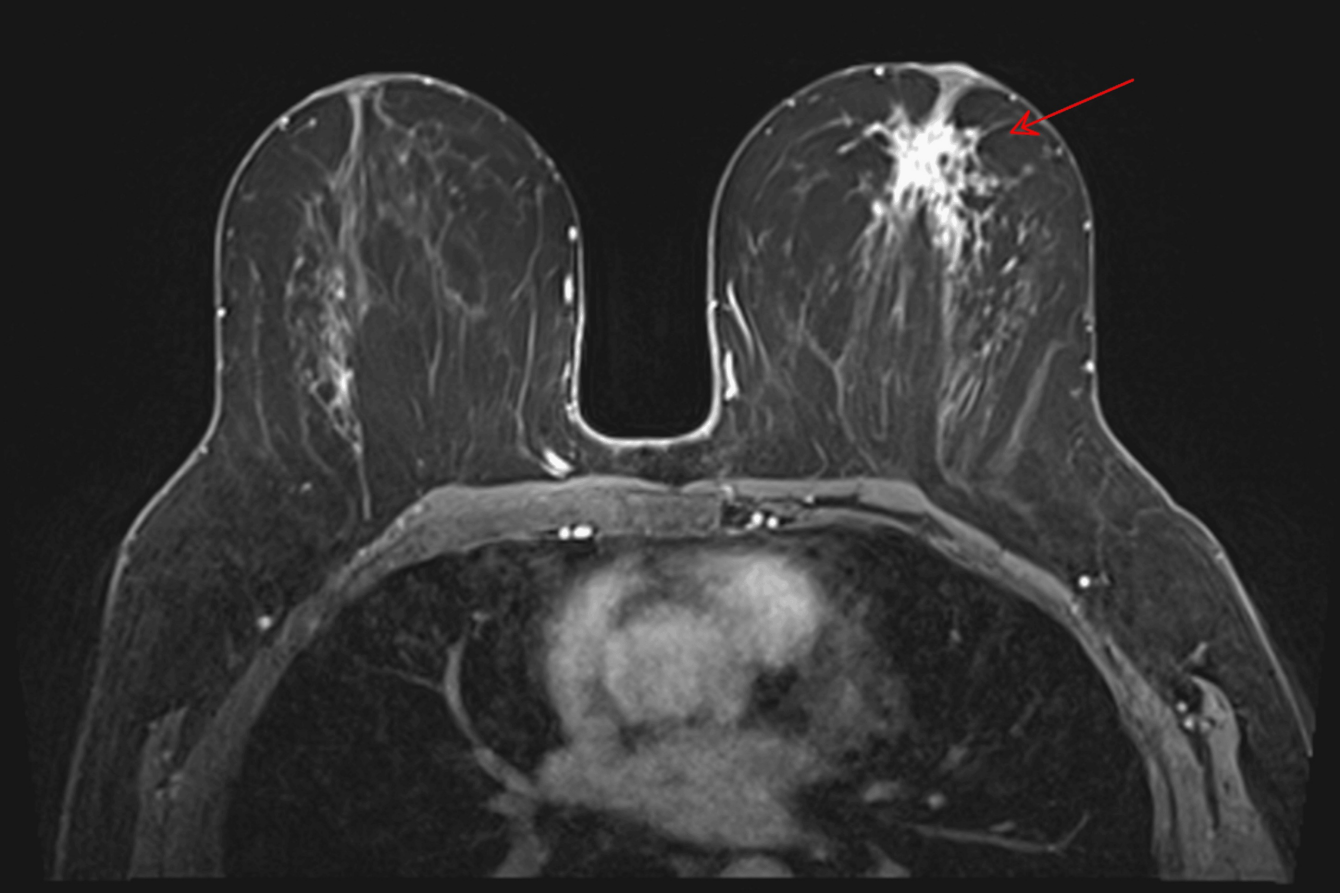
Breast MRI Non-mass enhancement in the upper retroareolar region of the left breast, with segmental distribution and spiculated margins, measuring 3.8 × 2.5 × 1.9 cm (red arrow). MRI: magnetic resonance imaging

**Figure 2:**
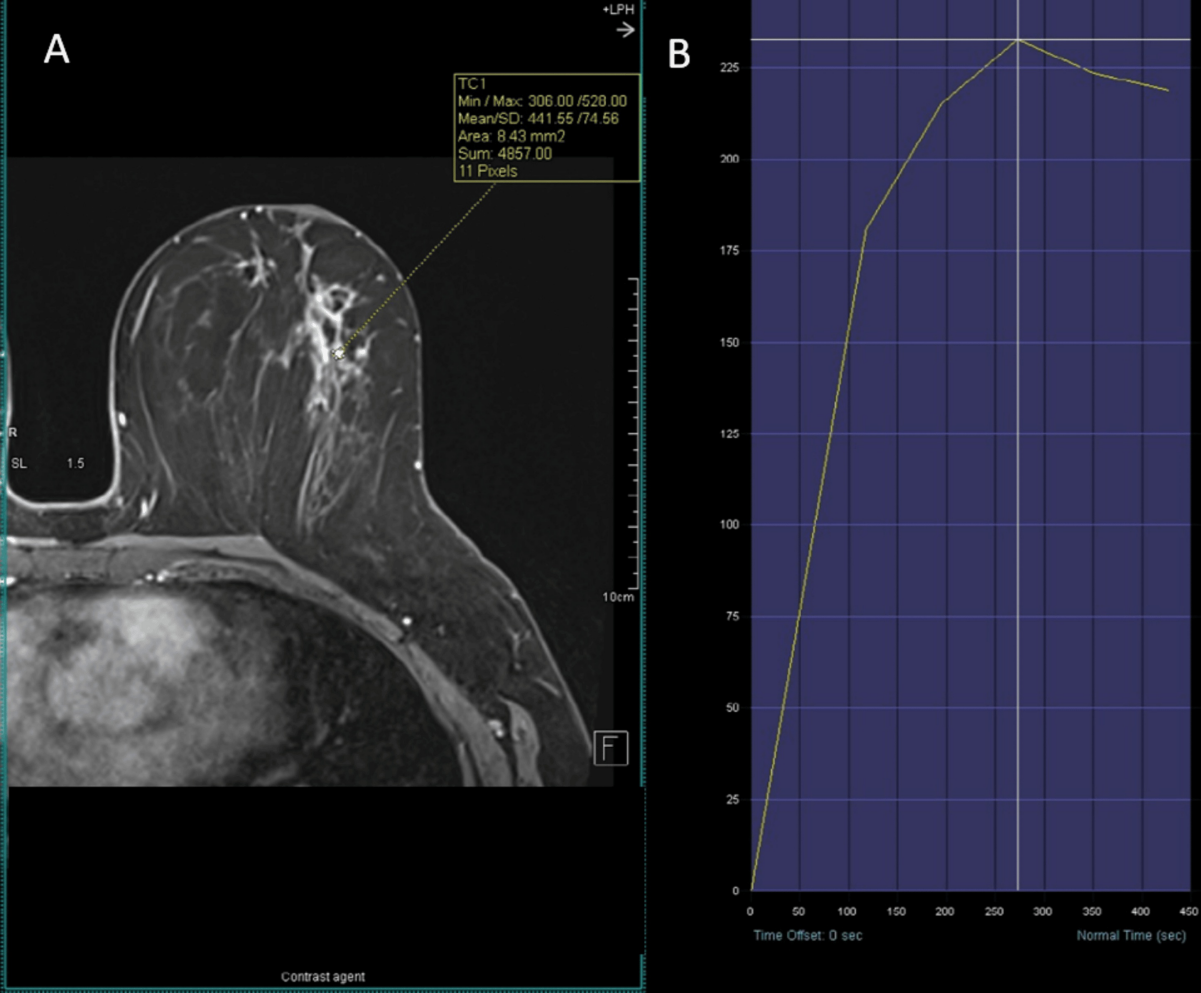
Breast MRI (DCE) A) DCE MRI with a representative region of interest for kinetic analysis. B) Type III (washout) kinetic curve. MRI: magnetic resonance imaging; DCE: dynamic contrast-enhanced

**Figure 3:**
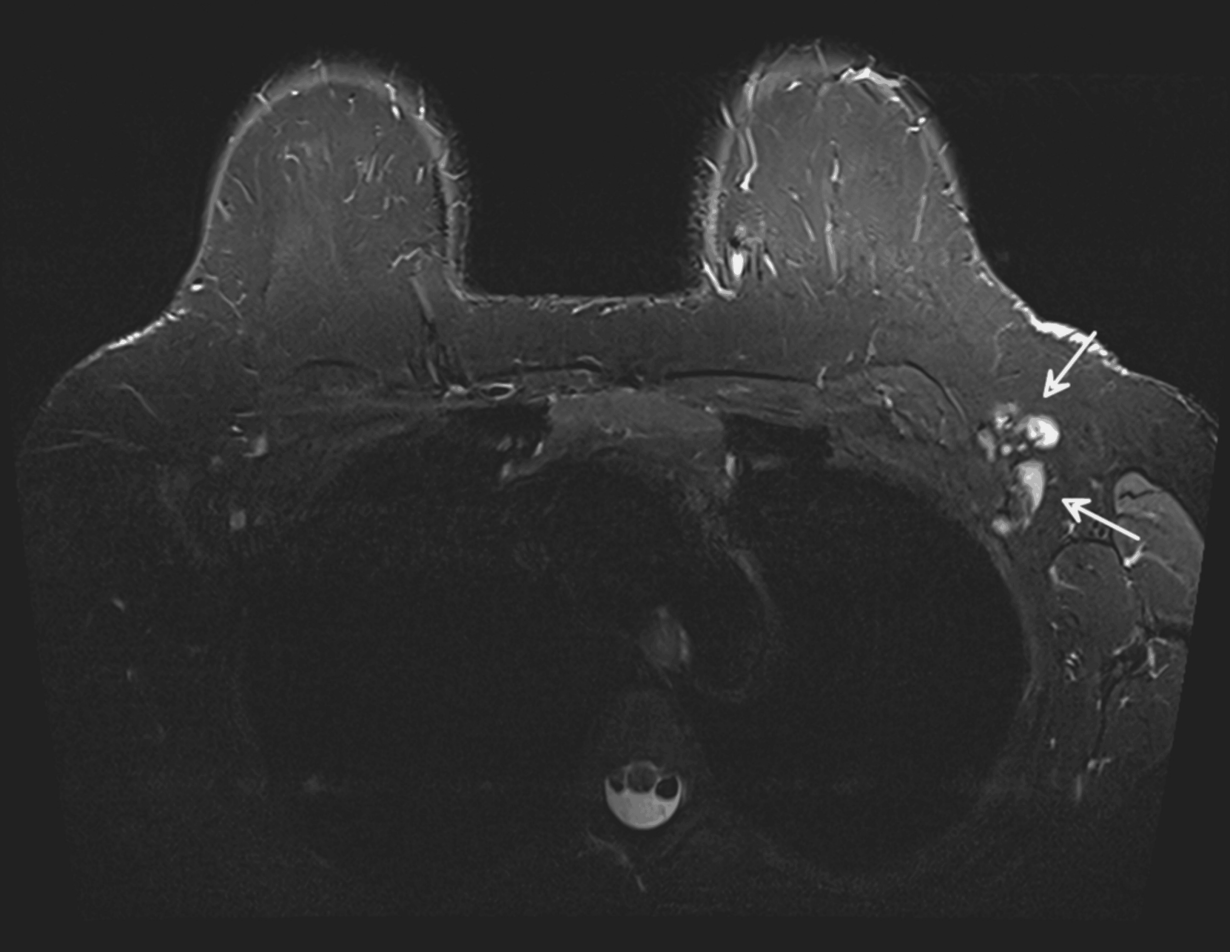
T2-weighted STIR breast MRI Axillary lymph nodes with cortical thickness up to 7 mm (white arrows). STIR: short tau inversion recovery; MRI: magnetic resonance imaging

A 54-year-old female presented to the breast clinic with a palpable mass in the central region of the left breast, confirmed on clinical examination. She was a non-smoker with no relevant risk factors, no history of lung disease, and no prior thoracic radiotherapy. Initial imaging with mammography and ultrasound was inconclusive. Breast magnetic resonance imaging (MRI) identified a unifocal area of non-mass enhancement in the upper retroareolar region of the left breast, with segmental distribution and spiculated margins, measuring 3.8 × 2.5 × 1.9 cm and classified as BI-RADS V (Figure 1) [7]. Dynamic contrast-enhanced (DCE) imaging demonstrated a representative region of interest for kinetic analysis (Figure 2A), yielding a type III (washout) kinetic curve (Figure 2B). T2-weighted short tau inversion recovery (STIR) sequences revealed four axillary lymph nodes with cortical thickening up to 7 mm (normal ≤3 mm), suspicious for metastatic involvement (Figure 3). 

A second-look ultrasound was performed, followed by ultrasound-guided core needle biopsy of the breast lesion and a suspicious axillary lymph node. Histopathological evaluation revealed invasive lobular carcinoma with marked nuclear pleomorphism and a relatively low proliferative index (Ki-67 approximately 10%), although this finding appeared discordant with the more aggressive features observed in the subsequent surgical specimen, likely reflecting intratumoral heterogeneity. Loss of E-cadherin expression on immunohistochemistry confirmed the lobular phenotype. The tumor was ER-negative, PR-negative, and HER2-negative (score 1+), consistent with a triple-negative phenotype. Lymphovascular invasion was present. Axillary lymph node biopsy confirmed metastatic involvement. Molecular analysis demonstrated activation of ERBB3-related PI3K-Akt-mTOR signaling and increased expression of angiogenesis- and proliferation-related markers. The molecular profile suggested in vitro resistance to taxanes and vinca alkaloids, with potential sensitivity to topoisomerase I inhibitors.

**Figure 4:**
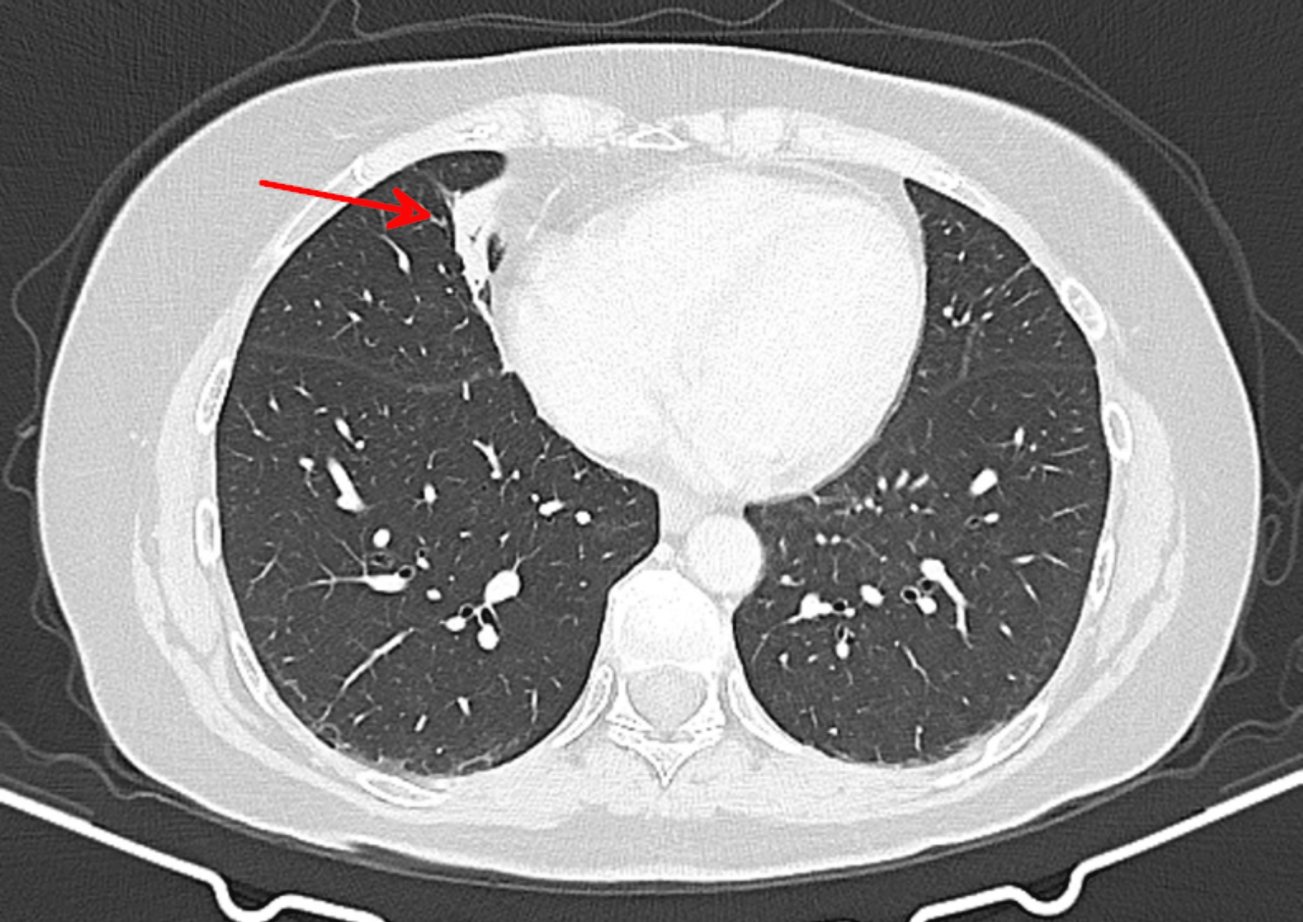
Baseline chest CT Subsegmental band-like atelectasis in the right middle lobe (red arrow). No suspicious pulmonary nodules or masses are identified. CT: computed tomography

Staging computed tomography (CT) scan of the thorax, abdomen, and pelvis, along with a bone scan, showed no evidence of distant metastases (M0). Chest CT demonstrated subsegmental band-like atelectasis in the right middle lobe, as well as the left breast lesion and small ipsilateral axillary lymph nodes (Figure 4). 

Routine laboratory tests, including white blood cell (WBC) count and liver and renal function, were within normal limits. According to the American Joint Committee on Cancer (AJCC) 8th edition, the disease was staged as cT2N+M0 [8]. Germline genetic testing, including BRCA1/2, revealed no pathogenic variants. Following multidisciplinary team (MDT) discussion, NACT was recommended. Although molecular profiling suggested potential reduced sensitivity to taxanes, standard-of-care taxane-based chemotherapy was initiated for TNBC.

A six-cycle weekly chemotherapy regimen was planned. The patient received intravenous paclitaxel 140 mg (60 mg/m²) and carboplatin 200 mg (AUC 1.5) weekly, with pembrolizumab 200 mg every three weeks starting from the fourth cycle. Epirubicin 90 mg/m² and cyclophosphamide 600 mg/m², along with pembrolizumab, were planned following restaging after completion of the sixth cycle in case of limited response. Prior to initiation of NACT, cardiac assessment with transthoracic echocardiography was normal.

During the first three weeks of treatment with paclitaxel and carboplatin, no major adverse events were observed. Laboratory tests were within normal limits, except for mild leukopenia (WBC 3,800/μL; reference range 4,000-11,000/μL) during the second cycle, which was managed with a single dose of granulocyte colony-stimulating factor (G-CSF). Pembrolizumab was not administered initially due to pending national authorization.

From the fourth cycle onwards, pembrolizumab was introduced and administered on the same day as paclitaxel and carboplatin chemotherapy. This represented the first and only exposure to pembrolizumab, which was subsequently discontinued due to treatment-related pulmonary toxicity.

Approximately 12 hours after this administration, the patient developed a fever up to 39.5°C, chills, fatigue, dry cough, and dyspnea. No rash, urticaria, or other cutaneous signs of hypersensitivity were observed. Physical examination revealed tachypnea with mild, non-specific auscultatory findings. Laboratory evaluation showed marked leukocytosis (WBC 26,780/μL), compared to 5,120/μL the previous day, without recent G-CSF administration. C-reactive protein (CRP) level was elevated at 21 mg/L (reference range <5 mg/L). Blood and sputum cultures were negative. This episode was initially considered a viral respiratory infection, as her children had concurrent viral illnesses. However, given the temporal association with therapy and the absence of microbiological evidence, immune-related pneumonitis was subsequently suspected. The patient was managed conservatively with supplemental oxygen (3 L/min), intravenous fluids, antipyretics, and close monitoring. No antibiotics were administered. Symptoms gradually resolved, and WBC normalized within four days, allowing discharge.

**Figure 5:**
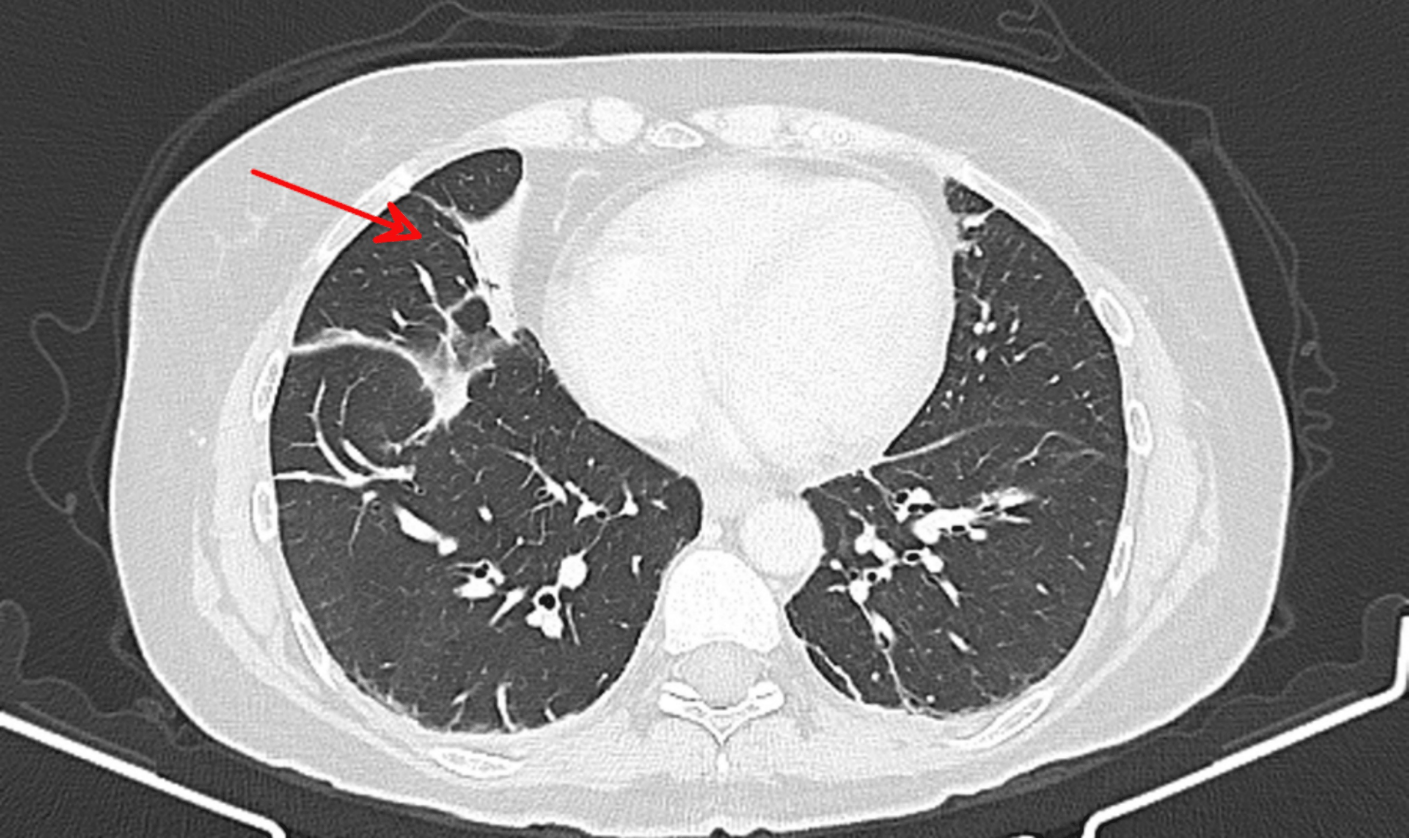
Chest CT 10 days after pembrolizumab administration Chest CT scan (lung window) shows an increase in band-like atelectasis involving the right middle lobe (red arrow). CT: computed tomography

**Figure 6:**
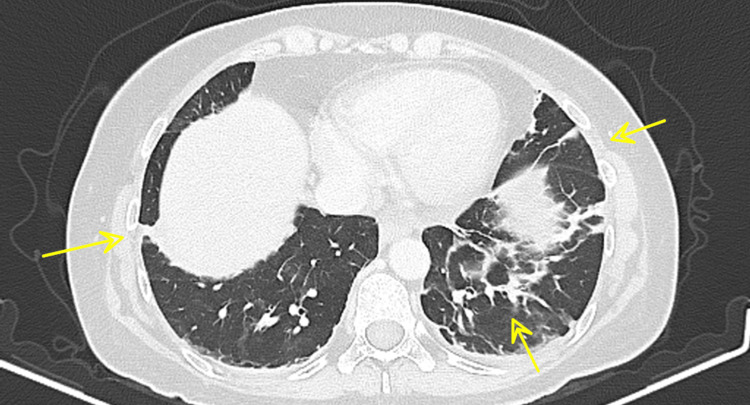
Chest CT 10 days after pembrolizumab administration Additional band-like atelectatic changes involving the lingula and both lower lobes (yellow arrows). CT: computed tomography

**Figure 7:**
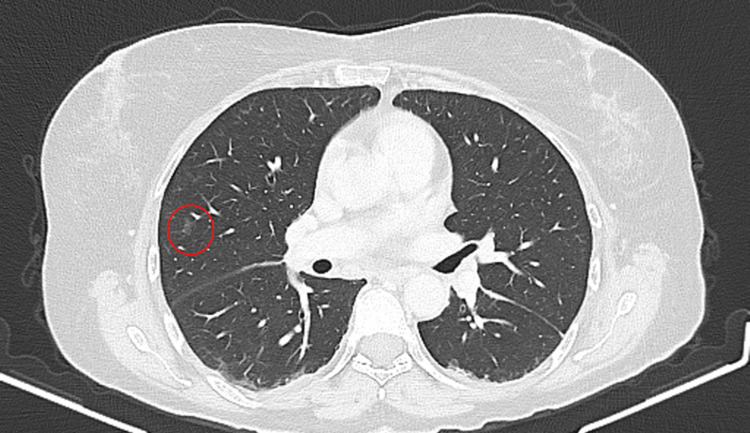
Chest CT 10 days after pembrolizumab administration An 8 mm ground-glass nodule in the right upper lobe (circle). CT: computed tomography

During the fifth cycle, the patient experienced a second episode of fever (up to 38.5°C) with cough a few hours after chemotherapy administration (paclitaxel and carboplatin). Auscultation revealed fine crackles over the lung bases. Leukocytosis (WBC 29,910/μL) was noted two days later, and CRP increased to 73.7 mg/L. CT imaging demonstrated progression of atelectasis involving the right middle lobe, lingula, and bilateral lower lobes; an 8 mm ground-glass nodule in the right upper lobe; and new bilateral trace pleural effusions (Figures 5-7).

The patient was hospitalized for five days and treated with intravenous fluids, supplemental oxygen (3 L/min), and empirical antibiotics (sultamicillin), with subsequent clinical improvement. A follow-up CT scan 20 days after administration of pembrolizumab showed near-complete resolution of atelectasis, resolution of the right upper lobe nodule and pleural effusions, but the appearance of a small pericardial effusion. Pleural and pericardial effusions were interpreted as potentially treatment-related inflammatory serositis versus less likely malignant involvement, given partial radiological resolution.

**Figure 8:**
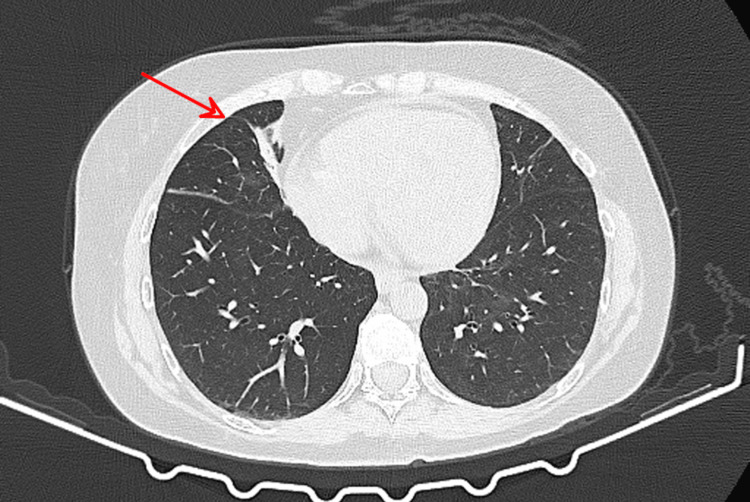
Follow-up chest CT 43 days after pembrolizumab administration Marked resolution of previously noted pulmonary abnormalities, with residual band-like subsegmental atelectasis in the right middle lobe (red arrow), representing a pre-existing finding. CT: computed tomography

During the sixth cycle, on the day of chemotherapy administration (paclitaxel and carboplatin), a third episode occurred, with more pronounced dyspnea and hypoxemia. CRP increased to 122 mg/L. Treatment included oral amoxycillin/clavulanate (1 g twice daily for 10 days), initiation of prednisolone 30 mg daily, and supportive care. The patient responded well, achieving complete clinical recovery within 10 days. Corticosteroids were gradually tapered over four weeks. Retrospective evaluation was consistent with grade 2 immune-related pneumonitis (CTCAE v6.0) [9]. A follow-up CT scan 43 days after immunotherapy demonstrated marked radiological improvement; the ground-glass nodule and residual atelectasis remained stable, while the pericardial effusion had decreased (Figure 8).

**Figure 9:**
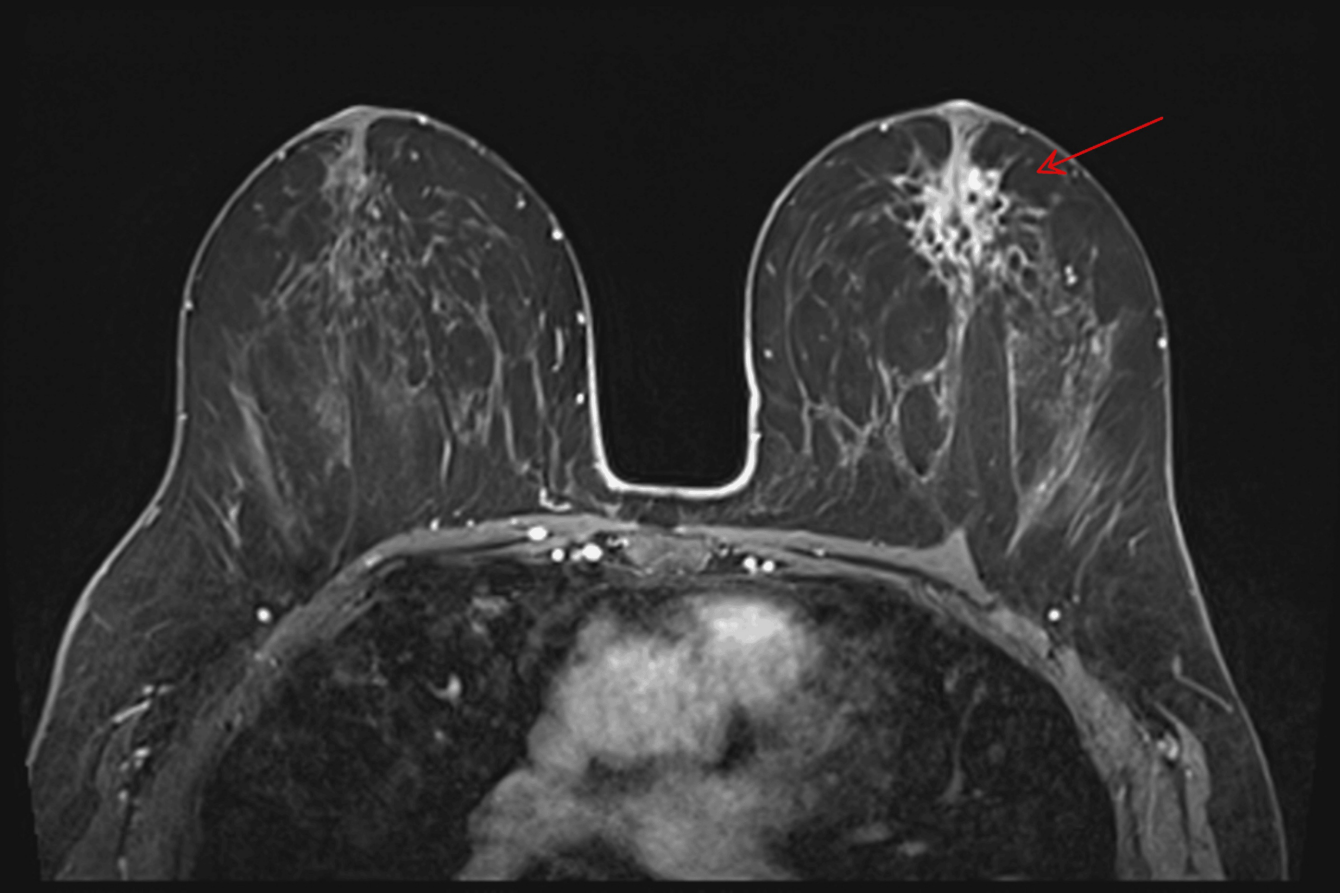
Post-neoadjuvant breast MRI The red arrow denotes suboptimal tumor response. MRI: magnetic resonance imaging

Given the suboptimal tumor response, as confirmed on post-treatment breast MRI (Figure 9), and recurrent treatment-related pneumonitis (one initial event followed by two recurrences), neoadjuvant therapy was discontinued. The patient subsequently underwent left mastectomy with level I/II axillary lymph node dissection five weeks later. Histopathological evaluation revealed grade III invasive lobular carcinoma (maximum diameter 4 cm) with associated lobular carcinoma in situ (LCIS), lymphovascular invasion, and perineural invasion. Surgical margins were clear. Nine of twelve lymph nodes were positive for macrometastases with extranodal extension. The pathological stage was ypT2N2aMx, with initial clinical staging cM0 (AJCC 8th edition).

The postoperative course was uneventful. She subsequently received four cycles of adjuvant chemotherapy with cyclophosphamide and doxorubicin, with no adverse events. She is planned to receive adjuvant radiotherapy to the axillary region and supraclavicular lymph nodes to reduce the risk of local recurrence. Clinical and radiological follow-up for respiratory monitoring will continue.

## Discussion

Respiratory complications are uncommon in the routine management of breast cancer and are typically observed in patients with pre-existing lung disease, metastatic involvement, or rare toxicities related to systemic therapy or radiotherapy. However, the increasing use of ICIs in TNBC has led to greater recognition of immune-related adverse events, particularly pneumonitis, which may significantly complicate treatment strategies in the neoadjuvant setting. In patients receiving chemoimmunotherapy, distinguishing immune-related pneumonitis from chemotherapy-associated pulmonary toxicity remains challenging due to overlapping clinical and radiological features and the absence of pathognomonic findings [10].

Immune checkpoint inhibitor-related pneumonitis (CIP) is an uncommon but potentially severe adverse event associated with PD-1 blockade. In breast cancer, its incidence remains low but clinically relevant, with reported rates of approximately 5% in patients treated with PD-1 inhibitors [11]. Clinical presentation is nonspecific, including dyspnea, cough, and hypoxemia. Chest CT plays a central role in diagnosis, as radiography is often nonspecific. The radiologic spectrum of CIP most commonly overlaps with patterns seen in interstitial lung disease, with organizing pneumonia representing the predominant pattern and nonspecific interstitial pneumonia also frequently observed. Less commonly, more severe presentations such as diffuse alveolar damage may occur. Imaging findings are heterogeneous, including ground-glass opacities, patchy or diffuse consolidations, interstitial infiltrates, and bilateral multilobar involvement. These patterns are not pathognomonic and require careful integration of clinical context, treatment exposure, temporal relationship to therapy, and exclusion of infection or disease progression [12].

Paclitaxel-induced pneumonitis, although rare, is a recognized complication of taxane-based chemotherapy. It typically presents with acute or subacute respiratory symptoms, including dyspnea, hypoxemia, and diffuse pulmonary infiltrates. Proposed mechanisms include hypersensitivity reactions, direct cytotoxic injury, and immune-mediated inflammation [13].

Carboplatin is a platinum-based chemotherapeutic agent whose toxicity profile is predominantly hematologic and is not associated with clinically significant pulmonary toxicity [14].

Infusion-related hypersensitivity reactions to paclitaxel or carboplatin were considered but deemed unlikely. Such reactions typically occur during or shortly after infusion and are often accompanied by cutaneous, bronchospastic, or hemodynamic manifestations. The delayed onset of symptoms and absence of systemic hypersensitivity features, together with the radiological pattern, argue against an acute infusion-related reaction [15].

From a differential diagnostic perspective, both immune CIP and taxane-associated pulmonary toxicity were considered. The evidence supporting an immune-mediated contribution includes the temporal relationship with the first and only administration of pembrolizumab and the rapid response to corticosteroid therapy. Conversely, recurrence of symptoms following paclitaxel re-exposure in the absence of further ICI administration supports a potential taxane-associated component. However, the overlapping timing of drug administration and combination chemoimmunotherapy precludes definitive attribution to a single agent.

Pneumonitis in this setting is more commonly associated with ICIs and taxane-based therapies [12]. Available evidence suggests that combining ICIs with taxanes does not significantly increase pneumonitis risk compared with immunotherapy alone, although interpatient variability remains considerable [6]. Overall, pneumonitis risk appears to vary across different ICI-based regimens but remains relatively low in combination therapy settings.

These findings highlight the considerable diagnostic challenge in distinguishing immune-related pneumonitis from chemotherapy-associated toxicity, given the absence of pathognomonic clinical or radiological features and the significant overlap between entities in patients receiving combination chemoimmunotherapy. The development of pleural and subsequent pericardial effusion during the clinical course raised the possibility of treatment-related inflammatory serositis versus malignant involvement. Although malignancy could not be definitively excluded, the temporal relationship with therapy and partial radiological improvement on follow-up imaging made a treatment-related inflammatory process more likely. Given the characteristic temporal association with treatment, compatible radiological findings, exclusion of infectious causes, and rapid clinical improvement following corticosteroid therapy, a clinical diagnosis of treatment-related pneumonitis was favored. Therefore, invasive diagnostic procedures such as bronchoscopy with bronchoalveolar lavage and pulmonary function testing were not performed, as they were not expected to alter acute management in a clinically stable patient.

This case describes an unusual presentation of suspected treatment-related pneumonitis during neoadjuvant chemoimmunotherapy, characterized by a hyperacute onset shortly after a single dose of pembrolizumab combined with chemotherapy. The patient initially received three cycles of paclitaxel and carboplatin without pulmonary toxicity. However, following the addition of pembrolizumab, a clinical picture consistent with pneumonitis developed, with subsequent recurrences after paclitaxel re-exposure despite the absence of further immunotherapy. 

To our knowledge, this represents an exceptionally early manifestation of CIP, which typically occurs weeks to months after treatment initiation, whereas taxane-induced pneumonitis usually develops within days to weeks [12,13]. Although early-onset immune-related adverse events have been described, particularly in patients with lung cancer, the rapid clinical course observed here remains highly atypical [16]. Rare cases of very early pneumonitis have been reported, supporting the possibility of accelerated immune activation in susceptible individuals [17].

An additional notable feature is the recurrence of pneumonitis following re-exposure to paclitaxel alone, suggesting a possible immune-mediated contribution to its pathogenesis. While this may reflect a sequential immune activation process, a direct chemotherapy-related pulmonary toxicity cannot be fully excluded. Similar patterns of persistent immune activation after ICI exposure have been previously described [18].

In summary, the temporal sequence and clinical evolution support a predominantly immune-mediated process. One possible explanation is that prior pembrolizumab exposure may have contributed to immune priming, thereby lowering the threshold for pulmonary toxicity upon subsequent exposure to paclitaxel. A synergistic interaction between ICI and chemotherapy has been proposed, although the exact mechanisms remain incompletely understood [19]. This interpretation is supported by the hyperacute onset, recurrence in the absence of further ICI exposure, and favorable response to corticosteroids, although overlapping drug-related toxicities cannot be entirely excluded.

In this context, clinical management was guided by suspected treatment-related pulmonary toxicity, based on the temporal relationship between drug exposure and symptom onset, severity, and recurrence pattern. Pembrolizumab was permanently discontinued and further neoadjuvant chemoimmunotherapy was withheld. Rechallenge with paclitaxel resulted in recurrent symptoms, leading to cessation of systemic therapy. Corticosteroids were administered during the second recurrence, resulting in rapid clinical improvement. The patient subsequently proceeded to definitive surgical management after stabilization. 

Of note, current American Society of Clinical Oncology (ASCO) and European Society for Medical Oncology (ESMO) guidelines for immune-related adverse events recommend early recognition of suspected pneumonitis, prompt exclusion of infectious causes, grading of severity, interruption of ICI therapy in symptomatic cases, and early initiation of corticosteroids for grade ≥2 toxicity [10,20].

Overall, this case illustrates the complexity of attributing pulmonary toxicity in patients receiving sequential or combination chemoimmunotherapy, where overlapping drug effects and nonspecific clinical and radiological patterns may limit definitive etiological classification. In such settings, clinical interpretation relies on integrated temporal assessment and multidisciplinary evaluation rather than isolated diagnostic features, highlighting the diagnostic uncertainty inherent in these clinical scenarios.

## Conclusions

Pneumonitis in patients receiving pembrolizumab-based chemoimmunotherapy for TNBC represents a rare but clinically significant complication. Differentiating immune-related pneumonitis from chemotherapy-induced pneumonitis, particularly taxane-associated toxicity, remains challenging due to overlapping clinical and radiological features. Early recognition, exclusion of alternative diagnoses, and prompt management are essential to optimize outcomes and guide treatment decisions regarding continuation or modification of therapy.
